# Modern Sociology

**Published:** 1896-10-31

**Authors:** 


### Modern Sociology.

### THE ARABIAN AND MOHAMMEDAN INFLUENCE.

"The brooding East with awe beheld
Her impious younger world,
The Roman tempest swelled and swelled,
And on her head was hurled.
The East bow'd low before the blast
In patient, deep disdain;
She let the legions thunder past,
And plunged in thought again."

These expressive words of Matthew Arnold well describe the state of the East before the advent of Mohammedanism. Like the power of water, the East is apparently unresisting, but at the same time it is irresistible. But the last act in that tremendous historical drama, which has been called "the huge duel between the East and the West," was about to commence. A cloud, as yet no bigger than a man's hand, was arising from the deserts of Arabia, which was destined to shake the newly-converted states which had arisen on the ruins of the old Western Empire to their foundations, and ultimately to destroy the effete Eastern Empire, which had been refounded on the shores of the Bosphorus, and to exert a permanent influence and power, which exists to this day, from Morocco to the frontiers of China and the islands of the Malay archipelago.

Comparatively little is known of the state of the Arab tribes before the Mohammedan era; but the little that is known is certainly of a favourable nature. Living a nomadic life from time immemorial, they were loosely knit together by the bonds of kinship, and the tradition of a common ancestry, but were almost devoid of any political organisation. Very proud of their race, very chivalrous in spite of their polygamy, but withal very "practical" in their ideas of, and their dealings with, women, much of the chivalry indeed which is associated with the Middle Ages of Christian Europe was really borrowed from them, as Mr. Wilfrid Blunt has shown in his recently-published work on the Arabs before the coming of Mohammed. Although fond of exulting in their noble descent, and keen genealogists, not only of themselves but also of their horses, they at the same time, like their near kinsmen the Hebrews, were an essentially democratic race; and it is this feature that gives to the history of Mohammedanism, apart from its religious aspect, an abiding and permanent interest to the student of sociology. Democracy is usually regarded as a product of Western ideas and culture, but really it is an heritage from the East—that is, from the nations of Semitic origin. Both Christianity and Mohammedanism are essentially democratic religions; not aristocratic, like the old Hellenic religion or the old Brahminical creed of India; and, again, not confined to one particular nation like the Hebrew, whose race and religion is indissolubly bound up together.

The famous text of the Koran (chap. v.): "O, true believers, surely wine, and lots, and images, and divining arrows are an abomination of the work of Satan; therefore avoid them that ye may prosper," expresses in the popular view the chief characteristics of the Mohammedan creed as distinct from the Jewish and the Christian. But Mohammedanism did something more than prohibit the drinking of wine, the making of images, and the practice of gambling. The aim of the Koran was to proclaim the eternal unity of God, and that the creed of His prophet Mohammed was the last and greatest of His revelations to mankind. To the student of sociology the nations who profess this creed are interesting, as showing its effect upon their social and civil institutions. One thing always to be remembered is that the Koran, unlike the Bible, is one book, the work of one man, and not the history of a nation through many centuries of its existence. Another difference, and, from a sociological point of view, a most important and interesting one, is that the Koran is full of directions as to the civil and political life of those who profess to believe in it. In this respect it bears some resemblance to the early books of the Old Testament, but is totally different to the New, which seems to avoid carefully all references to the political and social condition of the nations to whom the Gospel of Christianity was first preached. It has been said that slavery, war, and the gladiatorial games are not forbidden by the books of the New Testament, but no unprejudiced reader could venture to assert that the spirit of its teaching is not directly opposed to such institutions. On the contrary, the Koran and Mohammedanism uphold slavery and encourage war; in fact, both are inseparable from its teaching, as Christian and Jewish unbelievers must be reduced to pay tribute, and idolators must either become Mohammedans or must be put to the sword. Hence the difficulty which has arisen in all Mohammedan countries, sooner or later, where there are living races professing the Christian creed, of how to reconcile the progressive creed of Christianity with the stiff and unbending tenets of the Koran, especially where the rights and duties of unbelievers are concerned. A very intelligent observer, who had been travelling in the East, lately remarked that the Arab mind was stiff, legal, and formal, while the Persian, who represents what might be called the "Broad Church" of Mohammedanism, is imaginative and emotional. Historically, the ruling Mohammedan races have generally been more of the Arab type of character, and sometimes like the Turks of the present day, without the intellectual and moral qualities which for a brief period produced the bright and varied civilisation of Baghdad and of the Moors in Spain, where for some centuries the Arabs and their descendants were the masters of Europe in all intellectual progress, and the passers on to the world of mediæval Europe of the lost learning and philosophy of ancient Greece. But this intellectual ascendancy was very short-lived, and is not likely to recur again. A nation or a race, like an individual, must be judged by the ideal it places before itself. The ideal of Mohammedanism is universal domination over all races and creeds. The Caliphs, the successors of Mohammed, like the prophet himself, claimed supreme civil and spiritual power. The title, "Commander of the Faithful," implied an union of Emperor and Pope in the same person. This spirit still animates the more fanatical followers of Mohammed. It must be reckoned with by all interested in the future of sociology and, indeed, of humanity. Social progress only flourish in an atmosphere of peace. Mohammedanism is essentially a religion of war—arrogant, intolerant, and oppressive by its very nature to all non-believers. Yet it is the professed creed of the most restless and energetic of the races outside the Christian pale, with the possible exception of the Japanese, and as such must be dealt with by the sociologist.

